# Rapid enlargement of pulmonary benign metastasizing leiomyoma with fluid-containing cystic change: a case report

**DOI:** 10.1186/s40792-022-01444-3

**Published:** 2022-05-05

**Authors:** Takahiro Yanagihara, Naohiro Kobayashi, Tomoyuki Kawamura, Shinji Kikuchi, Yukinobu Goto, Hideo Ichimura, Yukio Sato

**Affiliations:** Department of Thoracic Surgery, Faculty of Medicine, University of Tsukuba, 1-1-1 Tennoudai, Tsukuba, Ibaraki 305-8575 Japan

**Keywords:** Pulmonary benign metastasizing leiomyoma, Benign metastasizing leiomyoma, Leiomyoma, Cystic change, Fluid-containing cyst, Pulmonary metastasis

## Abstract

**Background:**

Pulmonary benign metastasizing leiomyoma (PBML) is a rare disease that can occur in women with a history of uterine leiomyoma. Despite its benign histological features, like a malignancy, leiomyomas can on rare occasion spread to the lung. Typically, PBML presents with asymptomatic multiple solid lung nodules with slow tumor progression, following hysterectomy. Here, we present an atypical case with rapid enlargement of PBML with fluid-containing cystic change.

**Case presentation:**

We experienced a case of a 49-year-old woman with bilateral lung nodules following hysterectomy. Two nodules in the right lung had cystic change with fluid in the tumors. Hormone therapy was initiated after surgical biopsy of the left lung confirmed a diagnosis of PBML. However, the cystic component of right upper lobe lesion enlarged rapidly over the following 7 months, and, considering the risk of malignant transformation or tumor rupture, right upper lobectomy was performed. Pathologically, the fluid-containing tumor was diagnosed as PBML.

**Conclusion:**

Given the risk of rapid progression, we should carefully consider the surgical indications of fluid-containing PBML.

## Background

Pulmonary benign metastasizing leiomyoma (PBML) is a rare disease that can occur in women with a history of uterine leiomyoma. Leiomyomas infrequently spread to the lung like malignancy despite its benign histological features. Typically, PBML presents with asymptomatic multiple solid lung nodules with slow tumor progression, following hysterectomy. Here, we present a case with rapid enlargement of PBML with fluid-containing cystic change.

## Case presentation

**Fig. 1:**
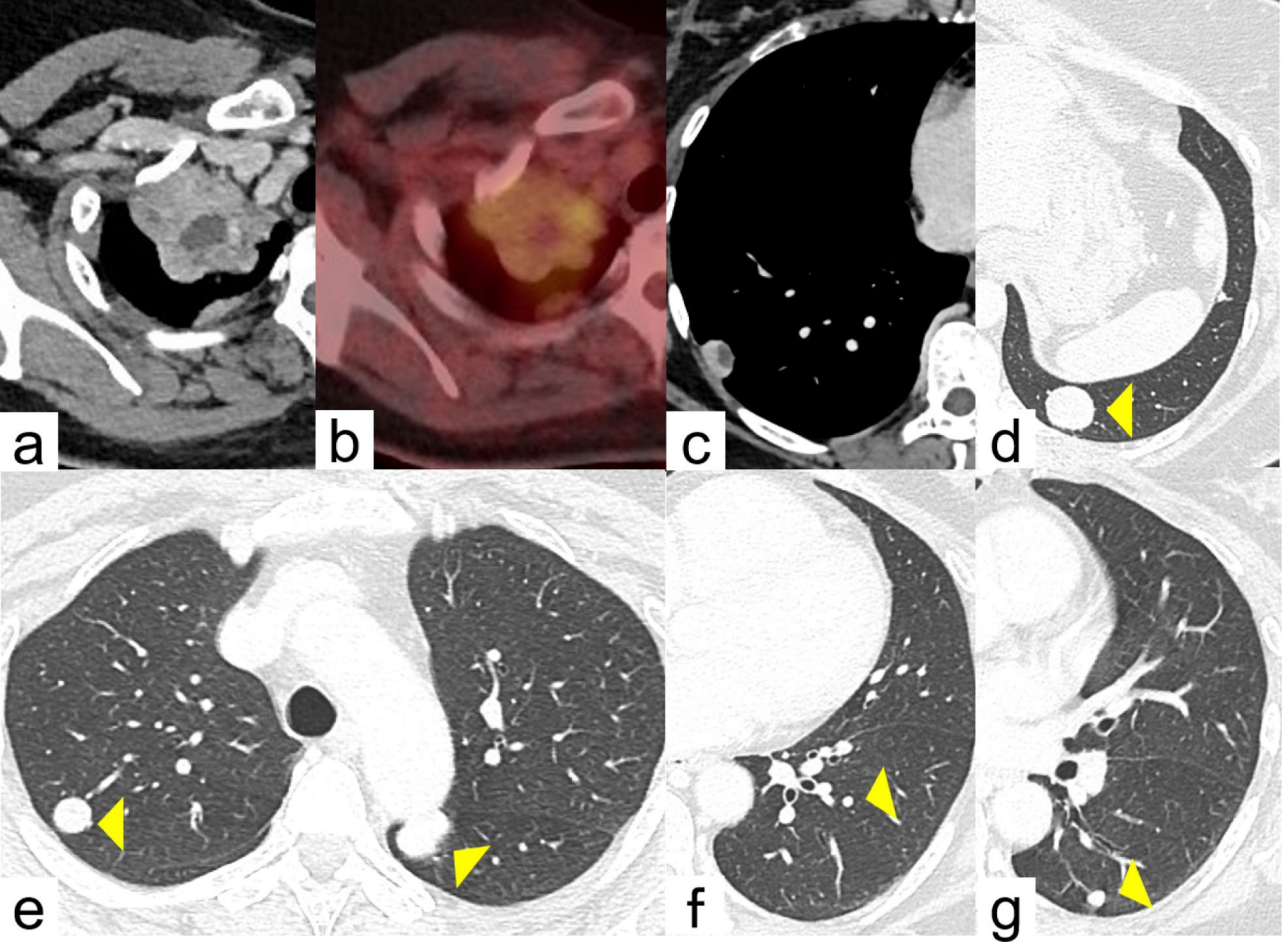
Computed tomography and positron emission tomography findings before the left lung biopsy. **a** The right upper lobe lesion contained a cystic fluid component. **b** Positron emission tomography revealed moderate avidity (maximum standardized uptake value, 4.93) in the right upper lobe lesion. **c** The tumor in the right lateral basal segment contained a cystic fluid component. **d**–**g** Solid tumors were located in the bilateral lungs (yellow arrowhead)

**Fig. 2:**
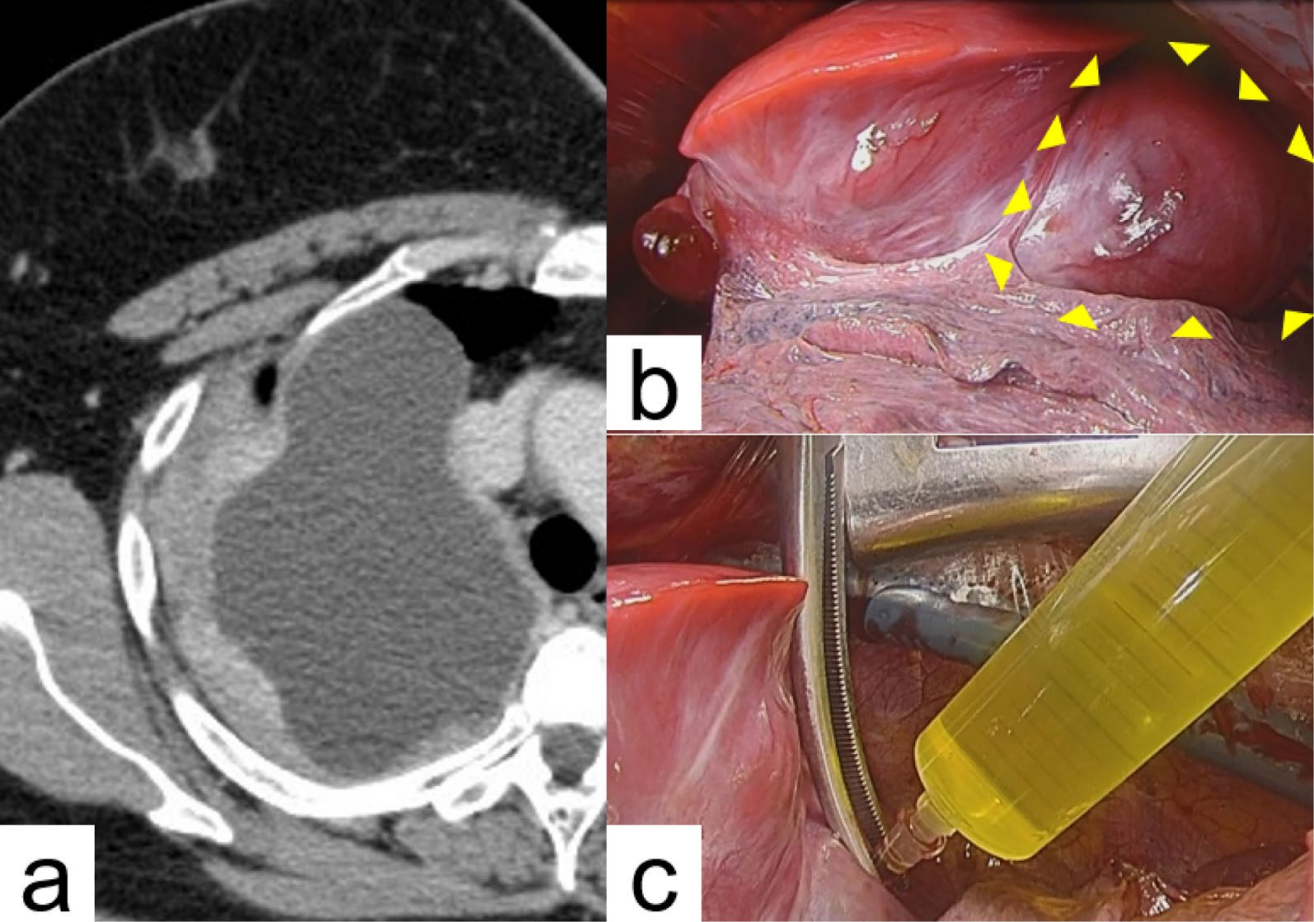
Preoperative computed tomography and intraoperative findings of rapid enlarged tumor in the right upper lobe. **a** The right upper lung lesion, especially cystic component, increased in size to 120 mm over a 7-month period. **b** The cystic component of the tumor, which had a dark-red surface, occupied most right upper lobe volume (yellow arrowheads). **c** Approximately 330 ml of serous fluid was egested from the tumor

**Fig. 3:**
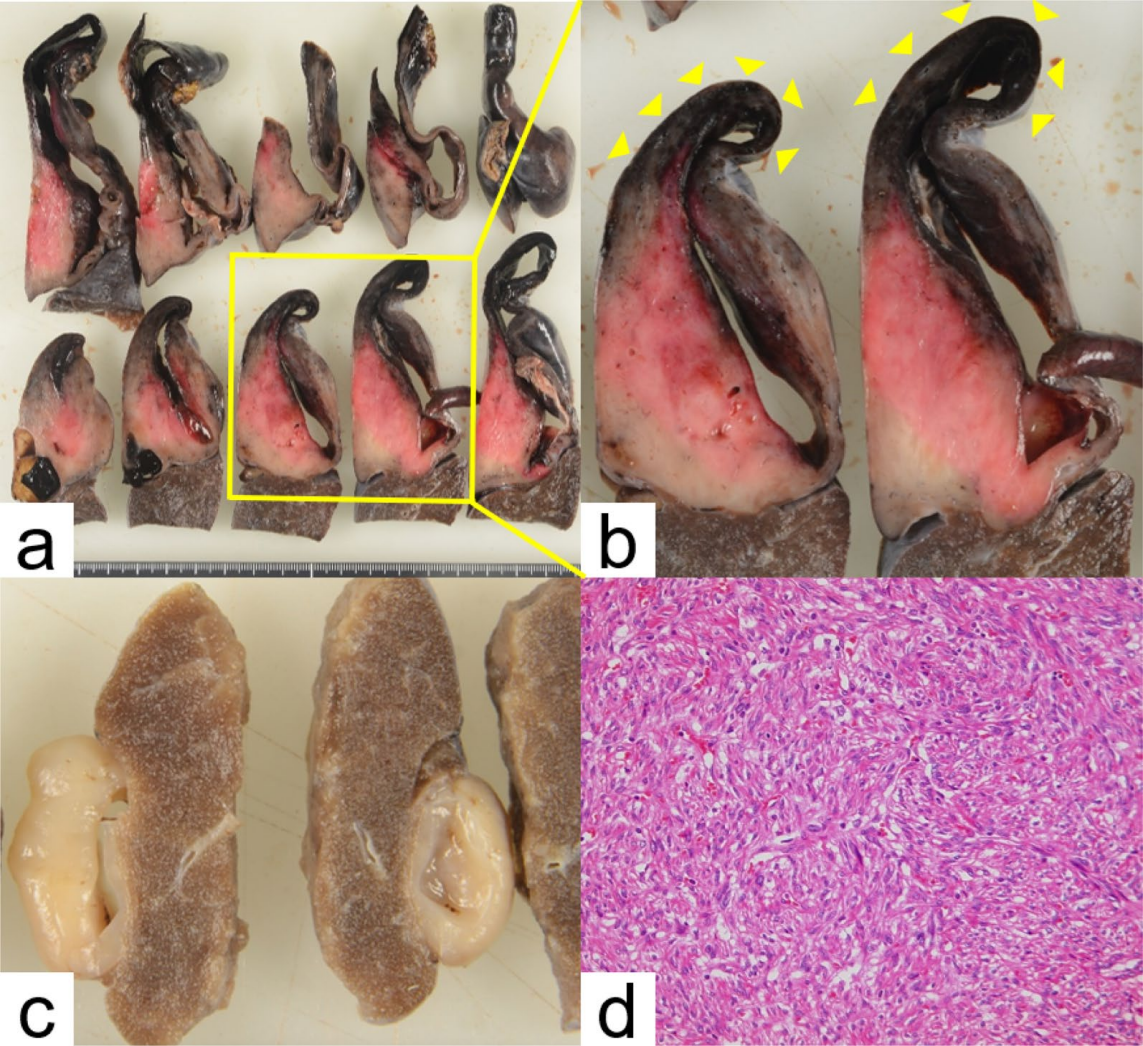
Pathological findings. **a** The macroscopic findings of right apical lesion. **b** The area of enlargement is indicated by the yellow square (**a**). The right upper lobe lesion included minute bleeding in the solid component around the cyst (yellow arrowheads). **c** Macroscopic findings of the right lateral basal segment lesion revealed no evidence of bleeding. **d** Microscopic findings revealed tangled spindle-shaped cells with little atypia and no tumor necrosis (original magnification ×200)

A 49-year-old woman, who had undergone hysterectomy and right oophorectomy for uterine myoma and ovarian cyst 9 years prior, visited her family doctor with a symptom of right chest pain. Physical examinations and laboratory data were normal. Computed tomography, however, showed multiple solid lung nodules in the bilateral lungs (Fig. 1). The maximal nodule size was observed in a 40-mm lesion in the right upper lobe (RUL) apex (Fig. 1a). Only two tumors, in the RUL apex and right lateral basal segment (S9), included a cystic fluid-filled component (Fig. 1a, c). Positron emission tomography revealed moderate avidity (maximum standardized uptake value, 4.93) only in the RUL apex lesion (Fig. 1b). Because multiple metastatic lung tumors of uterine leiomyoma were suspected, diagnostic wedge resection of the tumor in left lower lobe (Fig. 1d) under video-assisted thoracotomy was performed. The pathological findings were leiomyoma, similar to the original uterine myoma, and a definitive diagnosis of PBML was made. Following PBML diagnosis, treatment with medroxyprogesterone was initiated; however, the lesion in the RUL apex rapidly increased in size (especially the cystic component) from 40 to 120 mm over the subsequent 7 months (Fig. 2a). The lesion in the right S9, in contrast, remained at a size of 20 mm. Given the risk of rupture and malignant transformation of the tumors, right upper lobectomy and partial lung resection of the right S9 via lateral thoracotomy were performed. The large cystic tumor, which occupied most of the RUL volume, had a dark-red surface (Fig. 2b) and contained 330 ml of serous fluid (Fig. 2c). The right upper lobectomy was successful to excise the entire tumor. Examination of the cystic fluid showed a high level of CA125 (1199 U/ml). Pathologically, macroscopic findings revealed that the RUL tumor included minute bleeding in the solid component adjacent to the monolocular cystic change (Fig. 3a, b); however, there was no bleeding in right S9 cystic lesion (Fig. 3c). Microscopic findings showed tangled spindle-shaped cells with positive expression of α-smooth muscle actin, estrogen receptor, and progesterone receptor on immunohistochemical staining (Fig. 3d). There was little atypia and no tumor necrosis. Consequently, the cystic tumors were also diagnosed as PBML. Treatment with medroxyprogesterone was resumed and there was no evidence of new lesions or progression of residual lesion after 4 years of follow-up.

## Discussion

PBML is a rare disease that occurs in reproductive-age women with a history of uterine leiomyoma. The etiology of PBML is controversial. Previous reports have suggested three possibilities: (1) hormone-sensitive in situ proliferation of smooth muscle bundles; (2) benign smooth muscle cells transported from a uterine leiomyoma and colonized in the lung or metastasis of a low-grade leiomyosarcoma; and (3) surgically induced mechanical displacement from a preexisting uterine tumor [1].

Although PBML generally presents as solid and well-circumscribed lung nodules of various diameters, some atypical cases such as cystic tumor or military pattern have also been reported [2]. To the best of our knowledge, there have been only four cases of fluid-containing PBML (summarized in Table 1) [3–6].

**Table 1:** Reported cases of pulmonary benign metastasizing leiomyoma with fluid-containing cystic change

No	Authors	Age	Hysterectomy	Symptoms	Size (mm)	Contents	Pathology
1	Cotti et al. [3]	49	4 years prior	Fever	190	Brownish liquid and air	BML
2	Osadchy et al. [4]	49	2 years prior	Right neck swelling	130	Clear fluid	BML
3	Alimi et al. [5]	60	NA	Cough	NA	Fluid	Leiomyosarcoma
4	Song et al. [6]	61	1 year prior	Dry cough	101	Fluid	BML
5	Present case	49	9 years prior	Right chest pain	120, 20	Yellow fluid	BML

The diameter of the fluid-containing tumors in the four cases was 101–190 mm. Malignant transformation was found in one case out of four cases of fluid-containing tumor

*BML* benign metastasizing leiomyoma

Although malignant transformation of PBML has been reported in two cases previously, only one of them presented with fluid-containing tumor, which was concluded in the leiomyosarcoma (Table 1) [2, 6]. Moreover, the diameter of the fluid-containing tumors in the four cases was 101–190 mm, which tended to be larger than typical PBML. Fluid build-up in the tumor was thought to contribute to the increase of tumor size. In the previous four cases, not only was the mechanism ambiguous, the timing of the tumor size increase was also unclear, because these cases were already symptomatic at the time of detection. The present case differed from the previous cases in that we were able to observe the course of the tumor progression. Our experience with the present case suggests that fluid-containing PBML may have a latent risk of rapid tumor size increase and symptomatic lesions. In other words, we should carefully consider the surgical indications of fluid-containing PBML in light of the risk of malignant transformation and rapid enlargement. When operating on PBML, prioritizing the resection of fluid-containing lesions might be a valuable surgical consideration.

In the present case, hormonal therapy was administrated following pathological diagnosis (via left lung biopsy in consideration of multiple metastases) of PBML with positive expression of estrogen receptor and progesterone receptor. Hormone treatments were reported as an effective therapy for PBML with positive hormonal receptor [7, 8]. At the same time, with complete resection of all tumors being another possible treatment strategy, resection of the fluid-containing tumor in the right upper lobe arguably could have been performed instead of the left lung biopsy in the current case. As a result of our treatment strategy, the fluid-containing tumor in the right upper lobe showed rapid growth, while the residual non-fluid-containing tumors in the left lung remained stable for 4 years. These facts might add weight to the argument for surgical resection of fluid-containing PBML.

The rapid enlargement of uterine leiomyoma may result from infarction or cystic change in the leiomyoma. In addition, peri-nodular hydropic degeneration has occasionally been observed as a pathological feature [9]. However, only monolocular cystic change in the tumor was observed in the present case. We speculated two possible reasons for the rapid enlargement: (1) it was caused by fluid secretion into the tumor and (2) it resulted from intracystic hemorrhage. The fact that the fluid in the cyst was serous and contained a high level of CA125, which is generally related to uterine and ovarian disease, suggested the former to be more probable. Moreover, minute bleeding in the solid components was observed only in the RUL lesion, not in the right S9 lesion. Therefore, we speculated that this bleeding ensued secondarily to the rapid enlargement of cystic component of the tumor.

## Conclusions

This is a rare case of fluid-containing PBML with rapid tumor enlargement. Given the risk of rapid tumor progression, we should carefully consider the surgical indications of fluid-containing PBML.

## Data Availability

All data generated or analyzed during this study are included in this published article.

## Abbreviations

PBML: Pulmonary benign metastasizing leiomyoma
RUL: Right upper lobe
